# Supplementary material for: Macro CD5L+ deteriorates CD8+T cells exhaustion and impairs combination of Gemcitabine-Oxaliplatin-Lenvatinib-anti-PD1 therapy in intrahepatic cholangiocarcinoma
Source: Nat Commun. 2024 Jan 20;15:621. doi: 10.1038/s41467-024-44795-1 (PMC10799889; doi:10.1038/s41467-024-44795-1)
Supplement: Supplementary file 1 — Supplementary Information [file 41467_2024_44795_MOESM1_ESM.pdf]

Supplementary Information

Macro CD5L+ deteriorates CD8+T cells exhaustion and impairs combination of Gemcitabine-Oxaliplatin-Lenvatinib-anti-PD1 therapy in intrahepatic cholangiocarcinoma

Jia-Cheng Lu [1,4,5*], Lei-Lei Wu [3*], Yi-Ning Sun [3*], Xiao-Yong Huang [1, 4*], Chao Gao [4*], Xiao-Jun Guo [1,4,5], Hai-Ying Zeng [6], Xu-Dong Qu [7], Yi Chen [4], Dong Wu [8], Yan-Zi Pei [1,4,5], Xian-Long Meng [1,4,5], Yi-Min Zheng [1,4,5], Chen Liang [1,4,5], Peng-Fei Zhang [5], Jia-Bin Cai [1,4], Zhen-Bin Ding [1,4], Guo-Huan Yang [1,4], Ning Ren [1,4], Cheng Huang [1,4], Xiao-Ying Wang [1,4], Qiang Gao [1,4], Qi-Man Sun [1,4], Ying-Hong Shi [1,4], Shuang-Jian Qiu [1,4], Ai-Wu Ke [4,5], Guo-Ming Shi [1,2*#], Jian Zhou [1,4,5#], Yi-Di Sun [3#], Jia Fan [1,4,5#]

Affiliations

[1] Department of Liver Surgery and Transplantation, Zhongshan Hospital, Fudan University, Shanghai, 200032, China

[2] Clinical Research Unit, Institute of Clinical Science, Zhongshan Hospital of Fudan University, 200032, Shanghai, China.

[3] Institute of Neuroscience, CAS Center for Excellence in Brain Science and Intelligence Technology, Chinese Academy of Sciences, Shanghai 200031, China.

[4] Liver cancer Institute, Fudan University, Shanghai, 200032, China

[5] Key Laboratory of Carcinogenesis and Cancer Invasion, Ministry of Education of the People's Republic of China, Shanghai, 200032, China

[6] Department of Pathology, Zhongshan Hospital, Fudan University, Shanghai, 200032, China

[7] Department of Intervention Radiology, Zhongshan Hospital, Fudan University, Shanghai, China.

[8] Department of Radiology, Zhongshan Hospital, Fudan University, Shanghai, 200032, China.

[*] The authors contributed equally to this work

[#] Correspondence:

Jia Fan, Department of Liver Surgery and Transplantation, Zhongshan Hospital, Fudan University, Shanghai, 200032, China; Tel/Fax: +86 021 6404 1990; E-mail: fan.jia@zs-hospital.sh.cn

Yi-Di Sun, Institute of Neuroscience, CAS Center for Excellence in Brain Science and Intelligence Technology, Chinese Academy of Sciences, Shanghai 200031, China; Tel/Fax: +86 021 54921670; E-mail: ydsun@ion.ac.cn

Jian Zhou, Department of Liver Surgery and Transplantation, Zhongshan Hospital, Fudan University, Shanghai, 200032, China; Tel/Fax: +86 021 6404 1990; E-mail: zhou.jian@zs-hospital.sh.cn

Guo-Ming Shi, Department of Liver Surgery and Transplantation, Zhongshan Hospital, Fudan University, Shanghai, 200032, China; Tel/Fax: +86 021 6404 1990; E-mail: shi.guoming@zs-hospital.sh.cn

Supplementary Figures

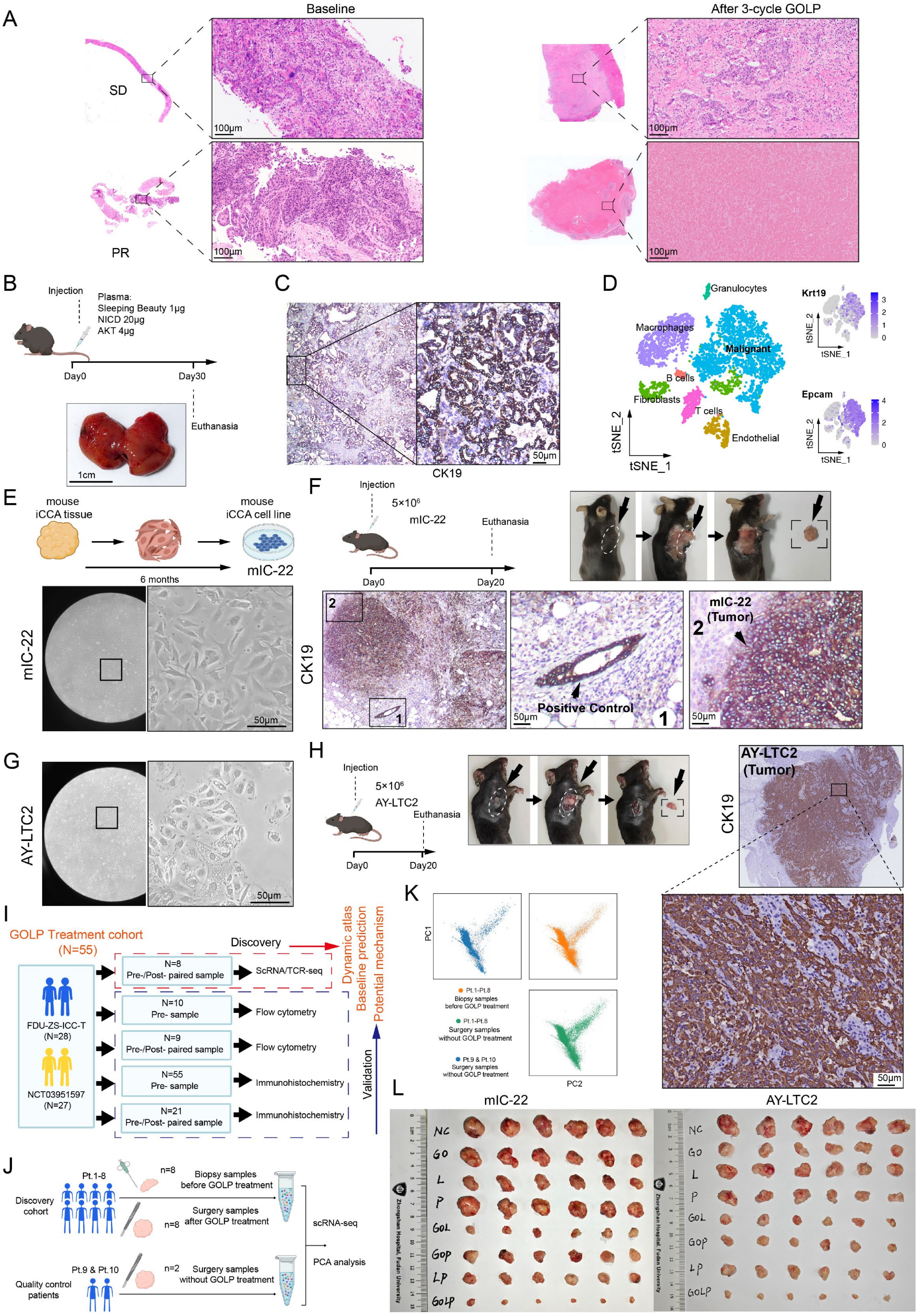

Supplementary Figure 1. Cohort design and scRNA-seq data quality control protocol

(A) Representative images of H&E staining of samples from iCCA patients after 3 cycles of GOLP treatment. Scale bar, 100μm.

(B) Workflow of mouse iCCA construction. Scale bar, 1cm. Created with BioRender.com.

(C) CK19 expression in the harvested liver of mouse with iCCA at Day30. Scale bar, 50μm.

(D) scRNA analysis of Krt19(CK19) and Epcam gene expression in the harvested liver of mouse with iCCA at Day30 (N=6,912 cells). Scale bar, 50μm.

(E) Workflow of the mIC-22 mouse iCCA cell line construction by our group. Scale bar, 50μm. Created with BioRender.com.

(F) Workflow of iCCA-bearing mouse with mIC-22 cell line and verification of CK19 expression in the subcutaneously implanted tumors. Scale bar, 50μm. Created with BioRender.com.

(G) Morphology of AT-LYC2 mouse iCCA tumor cells under high magnification microscope. Scale bar, 50μm.

(H) Workflow of iCCA-bearing mouse with AY-LTC2 cell line and verification of CK19 expression in the subcutaneously implanted tumors. Scale bar, 50μm. Created with BioRender.com.

(I) Cohort information and study design.

(J and K) PCA quality control of the scRNA-seq data from the 18 collected tumor tissues. Created with BioRender.com.

(L) Photos of tumor of mIC-22 or AY-LTC2 after different therapy strategies (the statistic results are shown in **Figure 1F**). Source data are provided as a Source Data file.

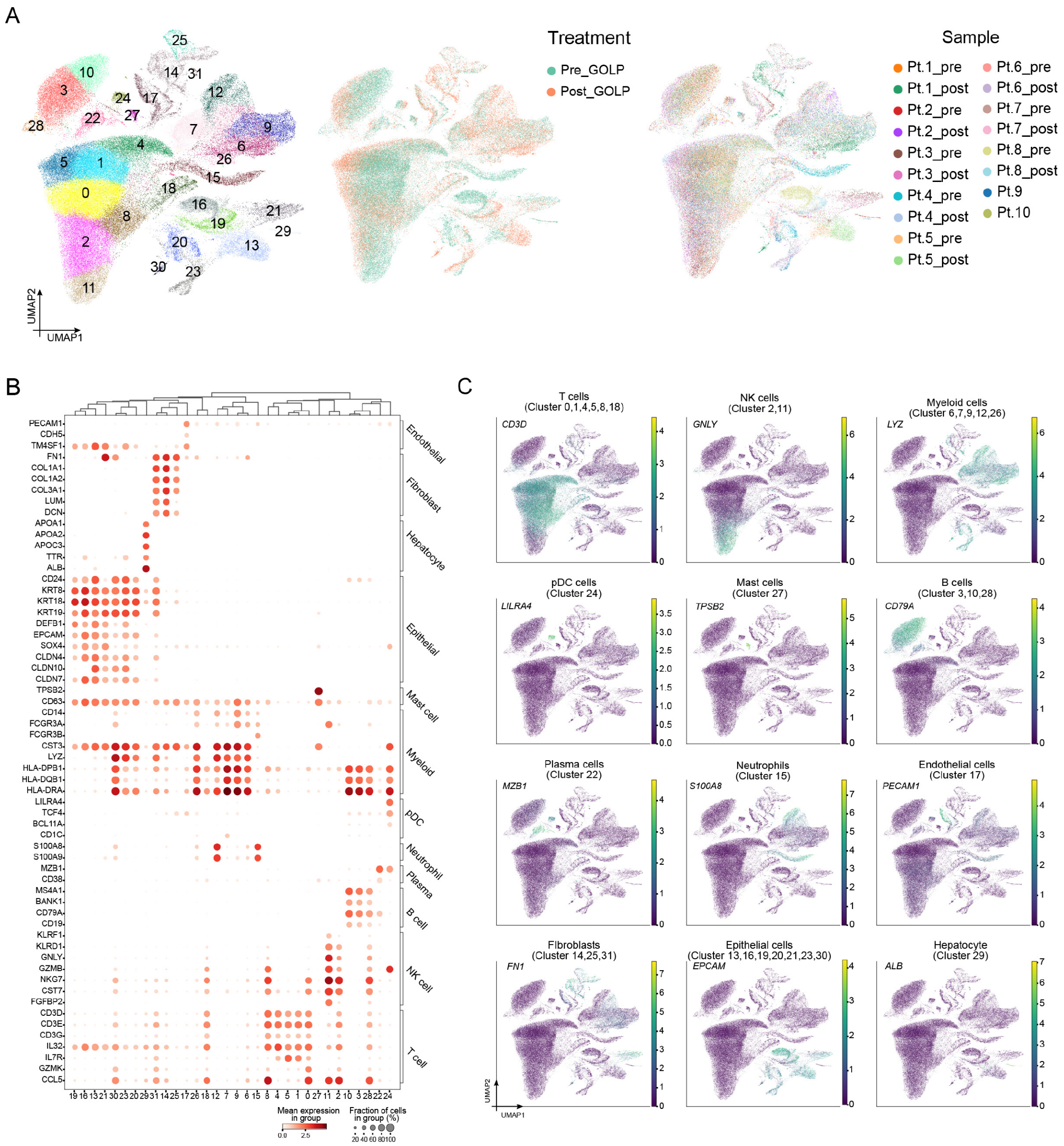

Supplementary Figure 2. Distinctive features of the main cell types

(A) UMAP analysis of all cells in the numbered clusters (Leiden algorithm). Treatment and sample type are projected on the same UMAP plot (N=131,139 cells).

(B) Cell-type marker gene expression in each numbered cluster.

(C) Feature plots of the main cell types (N=131,139 cells). Source data are provided as a Source Data file.

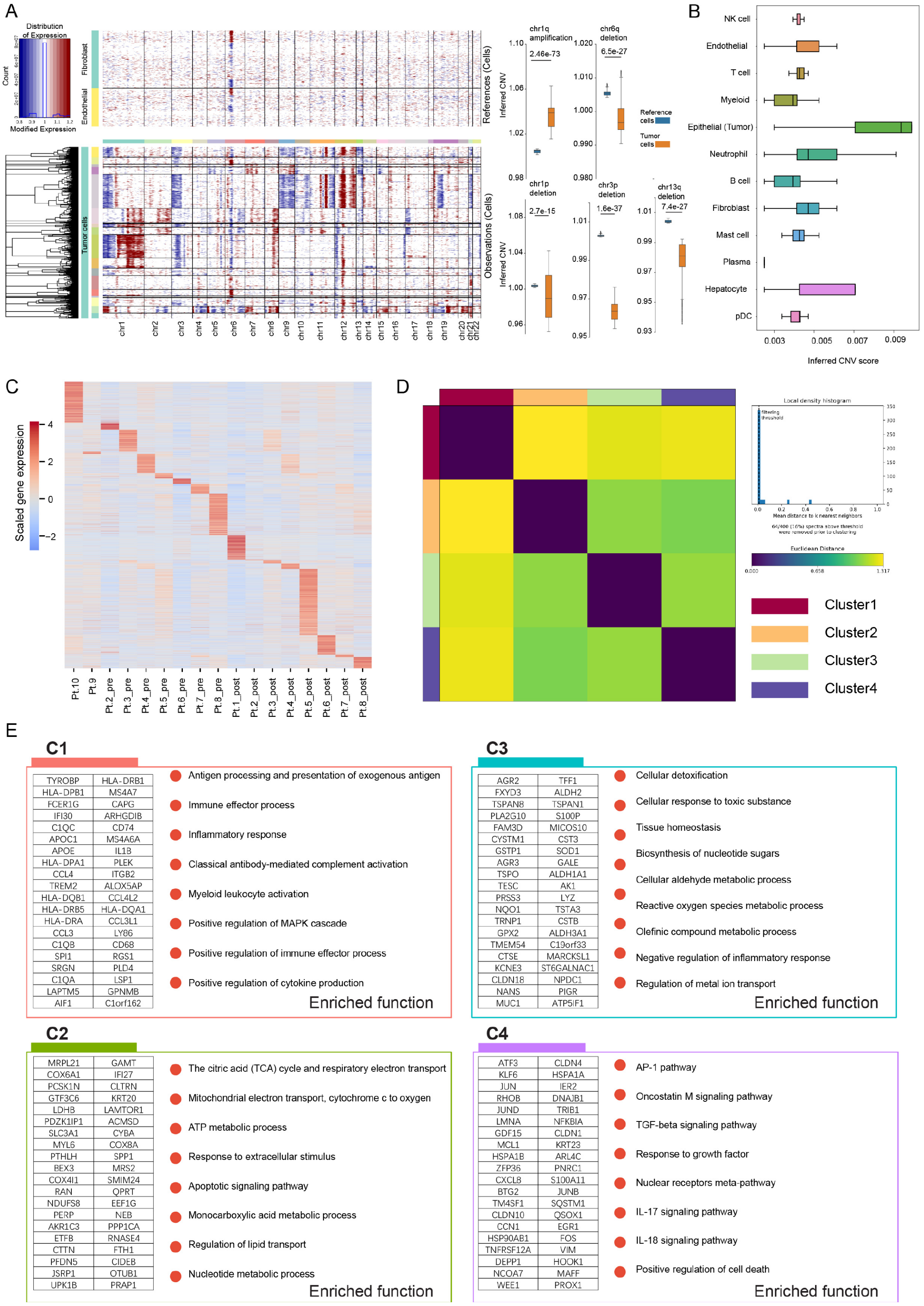

Supplementary Figure 3. Expression characteristics of tumor cells

(A) Inferred copy number variation of all tumor cells clustered by samples using endothelial cells and fibroblasts as references. Number of tumor cells= 14,411; Number of reference cells (endothelial cells and fibroblasts) = 6,507. The results are depicted in boxplots: center line indicates median, box represents

first and third quantiles, and whiskers indicate maximum and minimum values. Wilcoxon test, two-sided.

(B) CNV score inferred from the expression value of different cell types (N= 131,139 cells). Wilcoxon test, two-sided. The results are depicted in boxplots: center line indicates median, box represents first and third quantiles, and whiskers indicate maximum and minimum values.

(C) Heatmap of specific genes preferentially expressed in samples.

(D) Heatmap of the 4 meta-clusters established by cNMF with a distance outlier threshold of 0.1.

(E) Metagenes and enriched functions of the 4 different meta-clusters. Source data are provided as a Source Data file.

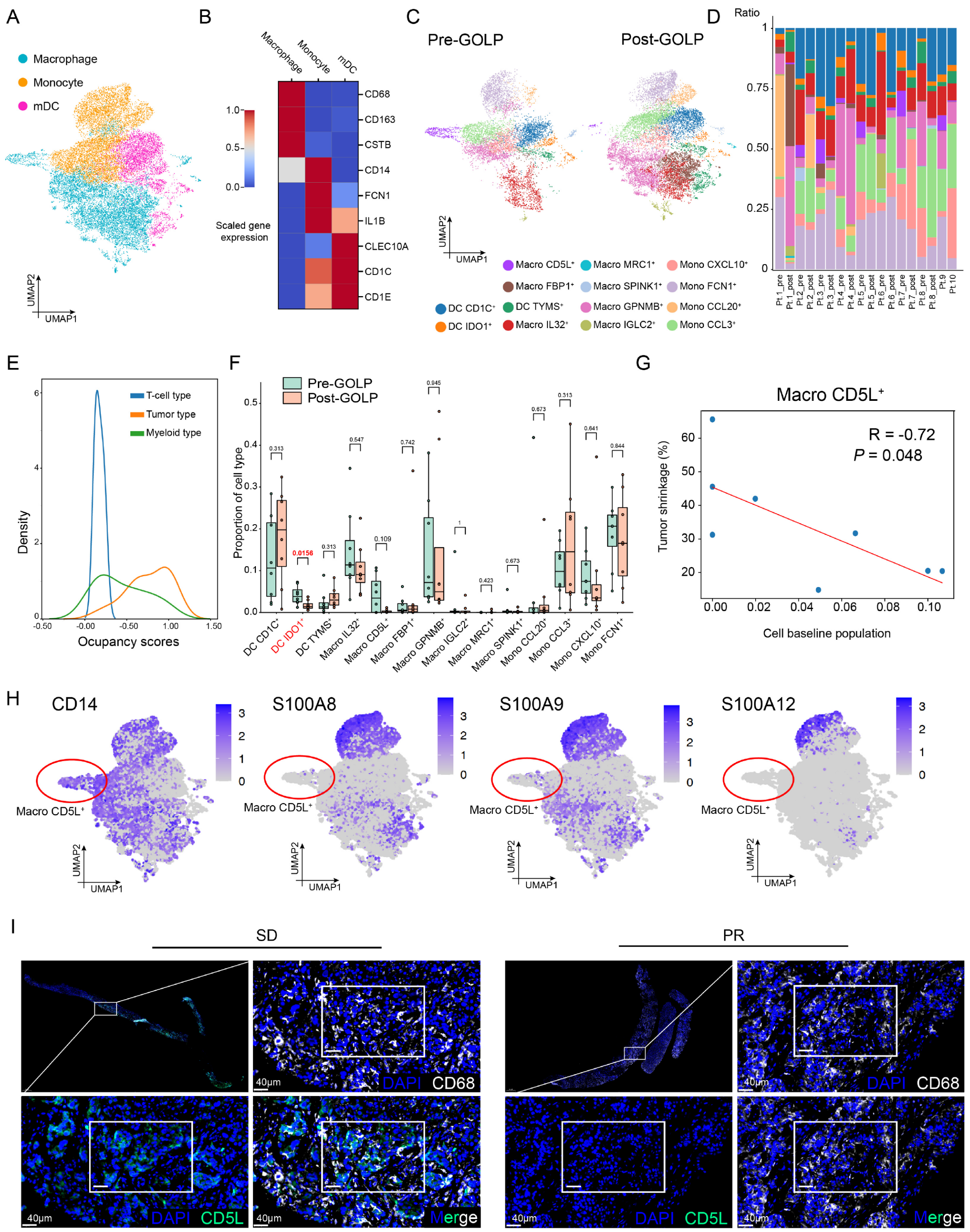

Supplementary Figure 4. Expression profiles of myeloid cells

(A) UMAP analysis of major subtypes of myeloid cells (N=20,825 cells).

(B) Matrix plot showing classic markers of macrophages, monocytes, and myeloid dendritic cells.

(C and D) UMAP plot and bar plot showing the minor subtypes of myeloid cells in each sample with high heterogeneity upon GOLP treatment (N=20,825 cells).

(E) Occupancy scores of T cell types, tumor cells and myeloid types.

(F) Pairwise comparison of myeloid subtype proportions between the pre-GOLP and post-GOLP groups.

N=8 independent patients, Paired Wilcoxon test, two-sided. The results are depicted in boxplots: center line indicates median, box represents first and third quantiles, and whiskers indicate maximum and minimum values.

(G) Relationship between tumor shrinkage and baseline proportion of Macro $CD5L^+$ (N=8 independent patients, Wald test, two-sided, $P = 0.048$, $R = -0.72$). Macro $CD5L^+$ demonstrated a general decreasing trend.

(H) UMAP Plot showing the expression of CD14 and the marker genes of MDSC (S100A8, S100A9, S100A12) in Myeloid clusters (N=20,825 cells).

(I) Original photos from Figure 4I showing representative samples from patients with SD and PR stained by IHC with anti-CD5L (green) and anti-CD68 (white) antibodies. Scale bar, 40μm. Source data are provided as a Source Data file.

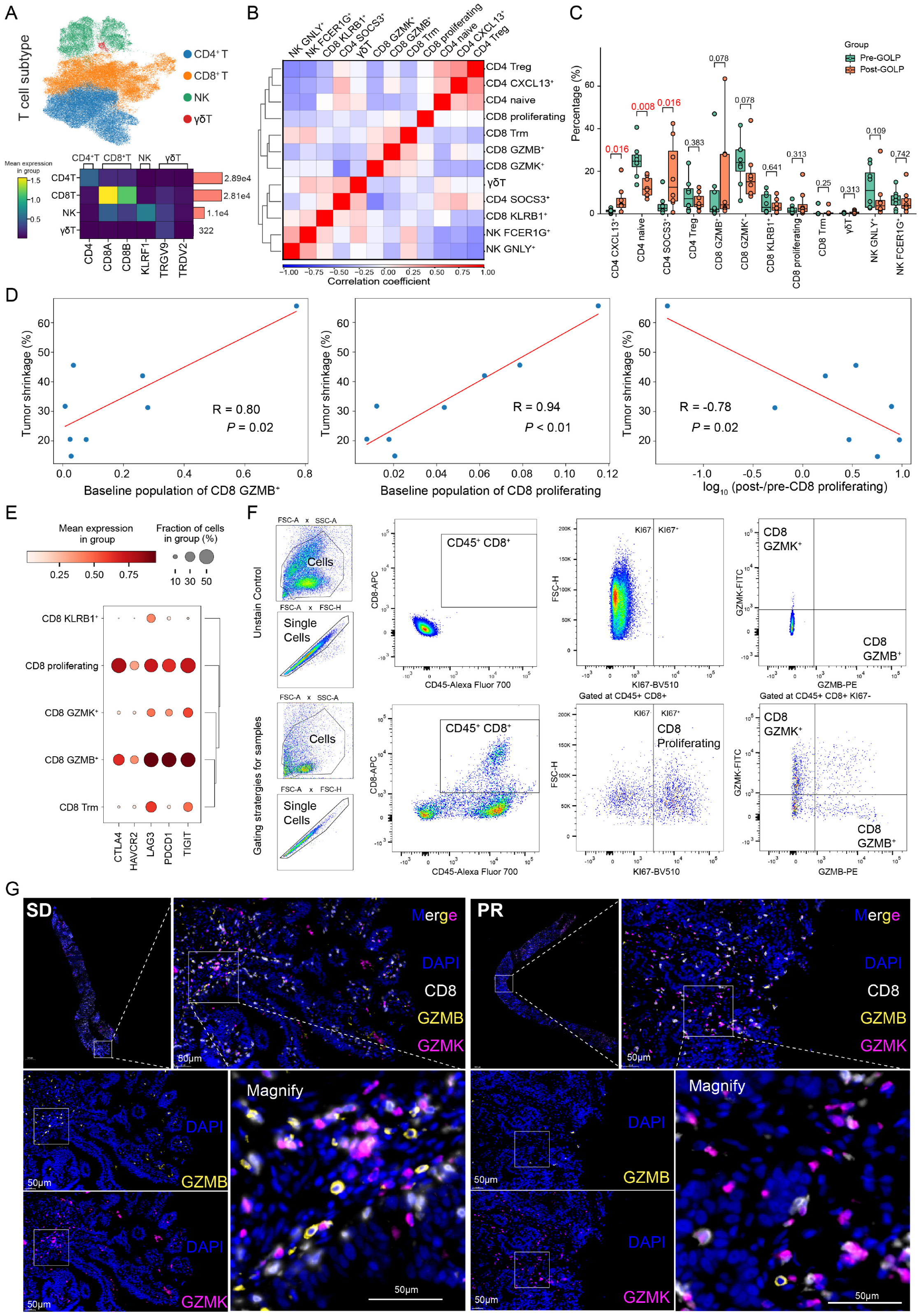

Supplementary Figure 5. T-cell subtypes and their characteristics

(A) Top: Major T-cell subtypes are projected on the UMAP plot (N=49,053 cells). Bottom: Classic markers of CD8$^+$ T cells, CD4$^+$ T cells, natural killer cells (NK cells) and γδT cells.

(B) Pearson's correlation analysis of T-cell subtypes confirmed the differences between T-cell subtypes.

(C) Changes in T-cell subtype populations pre- and post-GOLP treatment. N=8 independent patients. Paired Wilcoxon test, two-sided. The results are depicted in boxplots: center line indicates median, box represents first and third quantiles, and whiskers indicate maximum and minimum values.

(D) Relationship between tumor shrinkage and baseline proportions of CD8 GZMB$^+$ and CD8 proliferating or $\log_{10}$ normalized change in the proportion of CD8 proliferating after GOLP treatment (N=8 independent patients, Wald test, two-sided).

(E) Expression of exhaustion-related genes (CTLA4, HAVCR2, LAG3, PDCD1 and TIGIT) in CD8$^+$ T-cell subtypes.

(F) Flow cytometry gating strategies for CD8 GZMB$^+$, CD8 GZMK$^+$ and CD8 proliferating.

(G) Original photos from Figure 5K showing representative samples from patients with SD or PR stained by IHC with anti-CD8 (white), anti-GZMB (yellow) and anti-GZMK (purple) antibodies. Scale bar, 50μm. Source data are provided as a Source Data file.

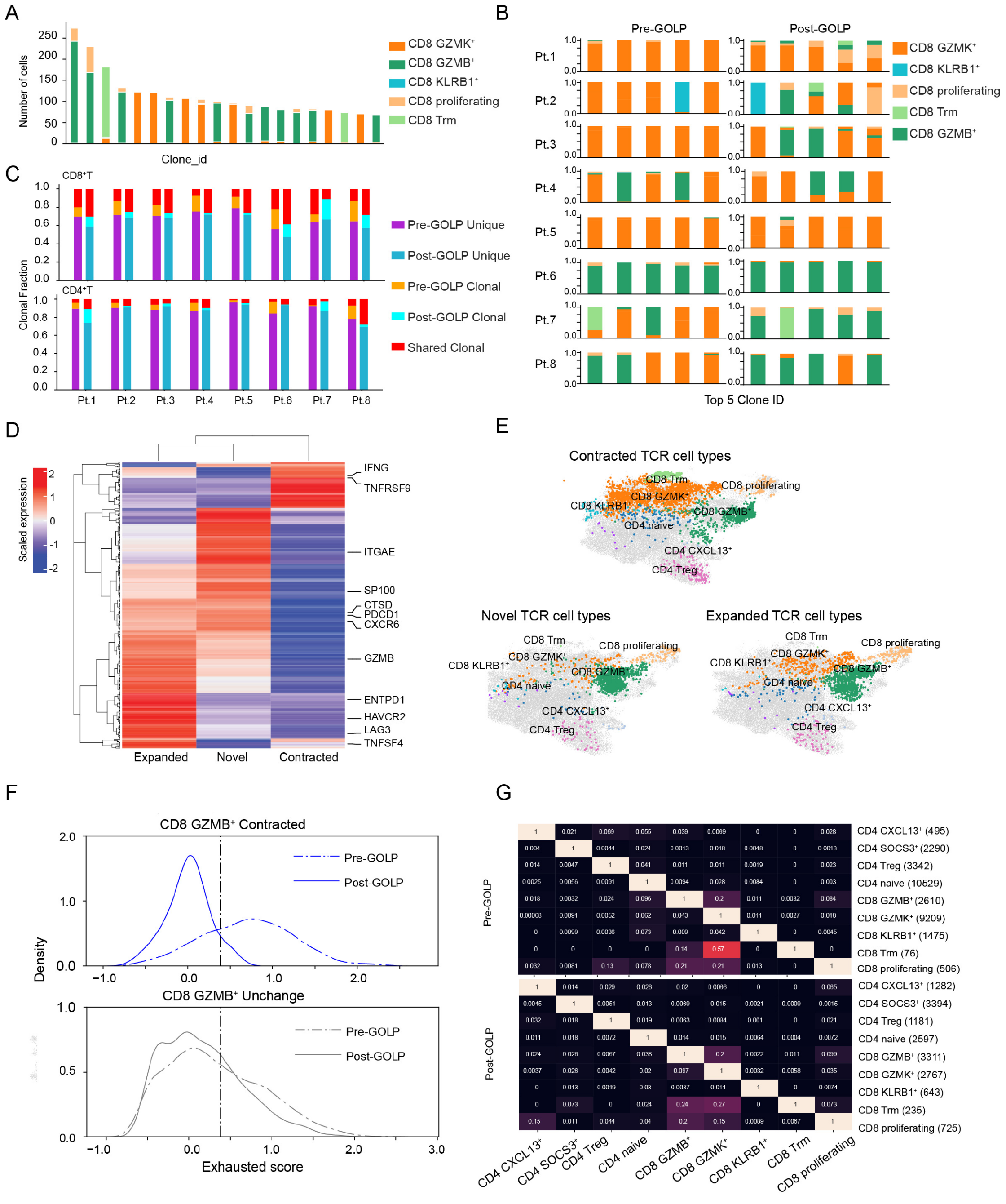

Supplementary Figure 6. Relationship between clonotype and phenotype

(A) Cell type distribution in the top clonotype IDs in all CD8$^+$ T cells (N=8 independent patients).

(B) Cell type distribution of the top 5 clone IDs in all CD8$^+$ T cells from paired post- /pre-GOLP samples in every patient (N=8 independent patients).

(C) Bar plot showing shared, unique, and clonal cell fraction changes in every paired sample (N=8 independent patients). The upper panel shows CD8$^+$ T cells, and the bottom panel shows CD4$^+$ T cells.

(D) Significantly enriched genes (N=328) in cells with contracted, novel and expanded TCR clones.

(E) UMAP plot showing the distribution of cell types with significantly contracted (N=2,972), novel

(N=1,356) and expanded (N=1,576) TCR clones.

(F) Exhausted score distribution in significantly Contracted CD8 GZMB$^+$ and Unchanged CD8 GZMB$^+$ in pre-GOLP and post-GOLP samples (N=8 independent patients). The gray dashed line represents the exhausted score cutoff, which was calculated from the exhausted scores of CD4 SOCS3$^+$ and CD4 naïve cells at a 99% confidence level (one-tail test).

(G) Fraction of TCR clone IDs shared among all cell types in pre-GOLP samples (upper panel) and post-GOLP samples (bottom panel) (N=8 independent patients). Source data are provided as a Source Data file.

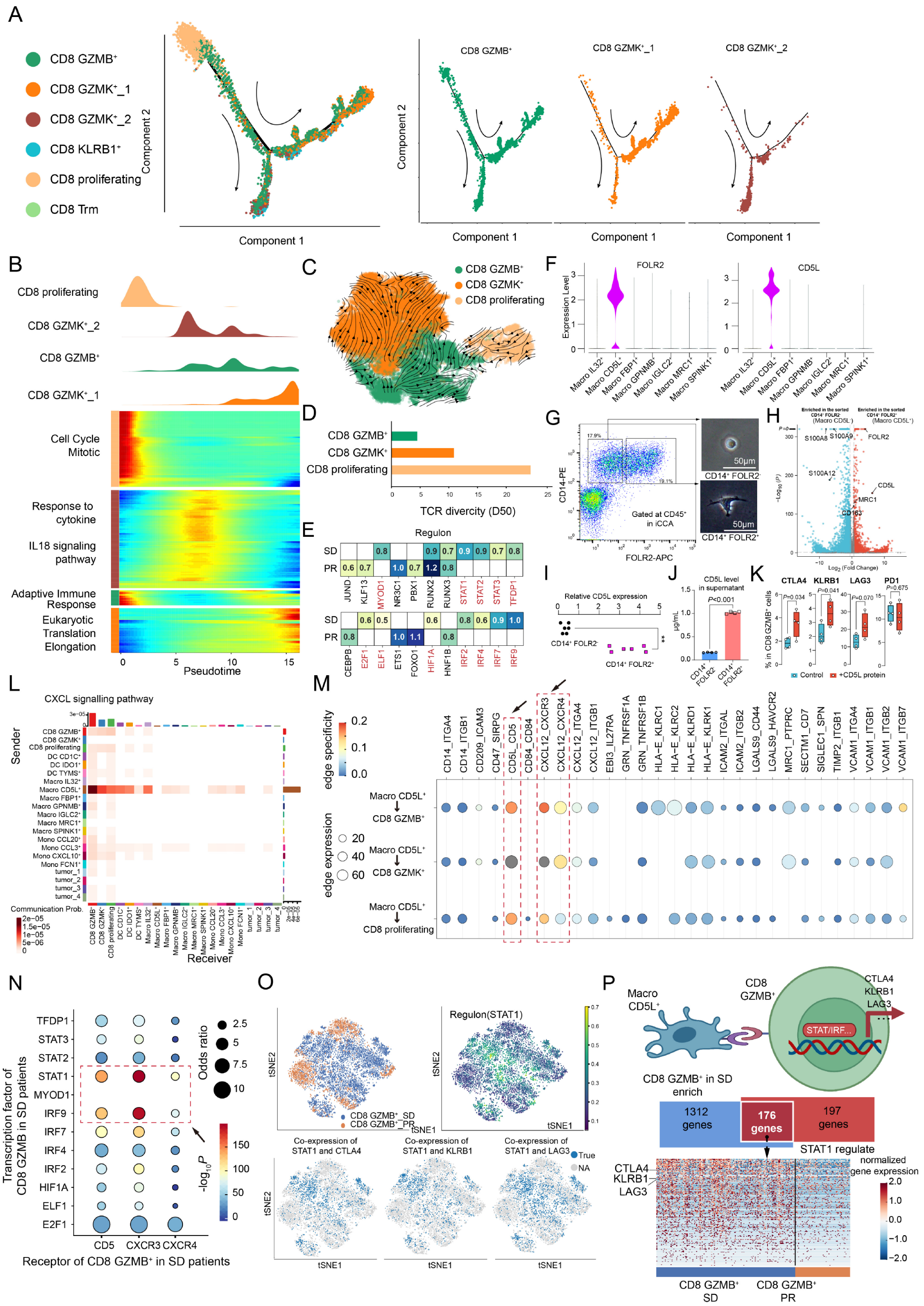

Supplementary Figure 7. CD8^{+} T-cell state transitions and key cell–cell interactions during GOLP treatment

(A) Cell trajectory analysis of CD8^{+} T cells showing the root position of CD8 proliferating, the plasticity of CD8 GZMB^{+}, and the terminal differentiation status of CD8 GZMK^{+} with two branch ends.

(B) Cell trajectory analysis showing that the three key $CD8^+$ subtypes could be categorized into four functional phases with different transcriptional states.

(C) RNA velocity profile of the three key $CD8^+$ T-cell subtypes (N= 19,128 cells).

(D) TCR diversity measured by D50 in the three key $CD8^+$ T-cell types.

(E) SCENIC analysis was used to predict transcription factors (TFs) regulating the downstream genes enriched in CD8 $GZMB^+$ in the PR and SD groups. TF regulon activities were quantified by AUCell.

(F) scRNA analysis showing the surface protein FOLR2 specifically expressed on Macro $CD5L^+$ across all the macrophages

(G) Sorting strategy of Macro $CD5L^+$ and Macro $CD5L^-$ in iCCA. Their cell morphology was shown on the right. Scale bar, 50μm.

(H) Volcano plot showing the differentially expressed genes between the sorted Macro $CD5L^+$ and Macro $CD5L^-$.

(I) CD5L expression in the sorted Macro $CD5L^+$ and Macro $CD5L^-$. (N=6 independent samples for each group, t test, two-sided, $**P < 0.01$).

(J) The sorted Macro $CD5L^+$ secreted significantly higher levels of CD5L protein than Macro $CD5L^-$ in the supernatants (N=4 independent samples for each group, t test, two-sided).

(K) $CD8^+$ T cells were cultured with conditioned media from Macro $CD5L^-$ with or without addition of CD5L protein (1μg/mL for 72h). Addition of CD5L protein significantly increased expression of the exhaustion marker CTLA4 on CD8 $GZMB^+$ cells (N=4 independent samples for each group, t test, two-sided).

(L) The communication probability analysis of CXCL signaling in the three $CD8^+$ T-cell subtypes, myeloid subtypes, and tumor meta-clusters (C1-C4).

(M) NATMI analysis showing the detailed cytokine communication between Macro $CD5L^+$ and CD8 $GZMB^+$, CD8 $GZMK^+$ and CD8 proliferating. The arrow indicates that the Macro $CD5L^+$–CD8 $GZMB^+$ interactions were mainly achieved by CD5L-CD5 and CXCL12-CXCR3/4.

(N) Co-expression of three receptor genes (CD5, CXCR3 and CXCR4) with enriched TFs of CD8 $GZMB^+$ in SD patients.

(O) TSNE plot (N=5,610 cells) showing the distribution of STAT1 expression and STAT1 co-expression with its downstream regulated genes (CTLA4, LAG3 and KLRB1) in CD8 $GZMB^+$ from patients with SD and PR.

(P) Summary of the potential crosstalk between Macro CD5L$^+$ and CD8 GZMB$^+$. Cytokine pathways (CXCL12-CXCR3/4 and CD5L-CD5) activate transcription factors (such as STAT family and IRF family transcription factors), ultimately upregulating downstream exhaustion markers (such as CTLA4 and LAG3) in CD8 GZMB$^+$. Right: heatmap showing the common genes enriched in CD8 GZMB$^+$ (SD patients) and regulated by STAT1. Created with BioRender.com. Source data are provided as a Source Data file.

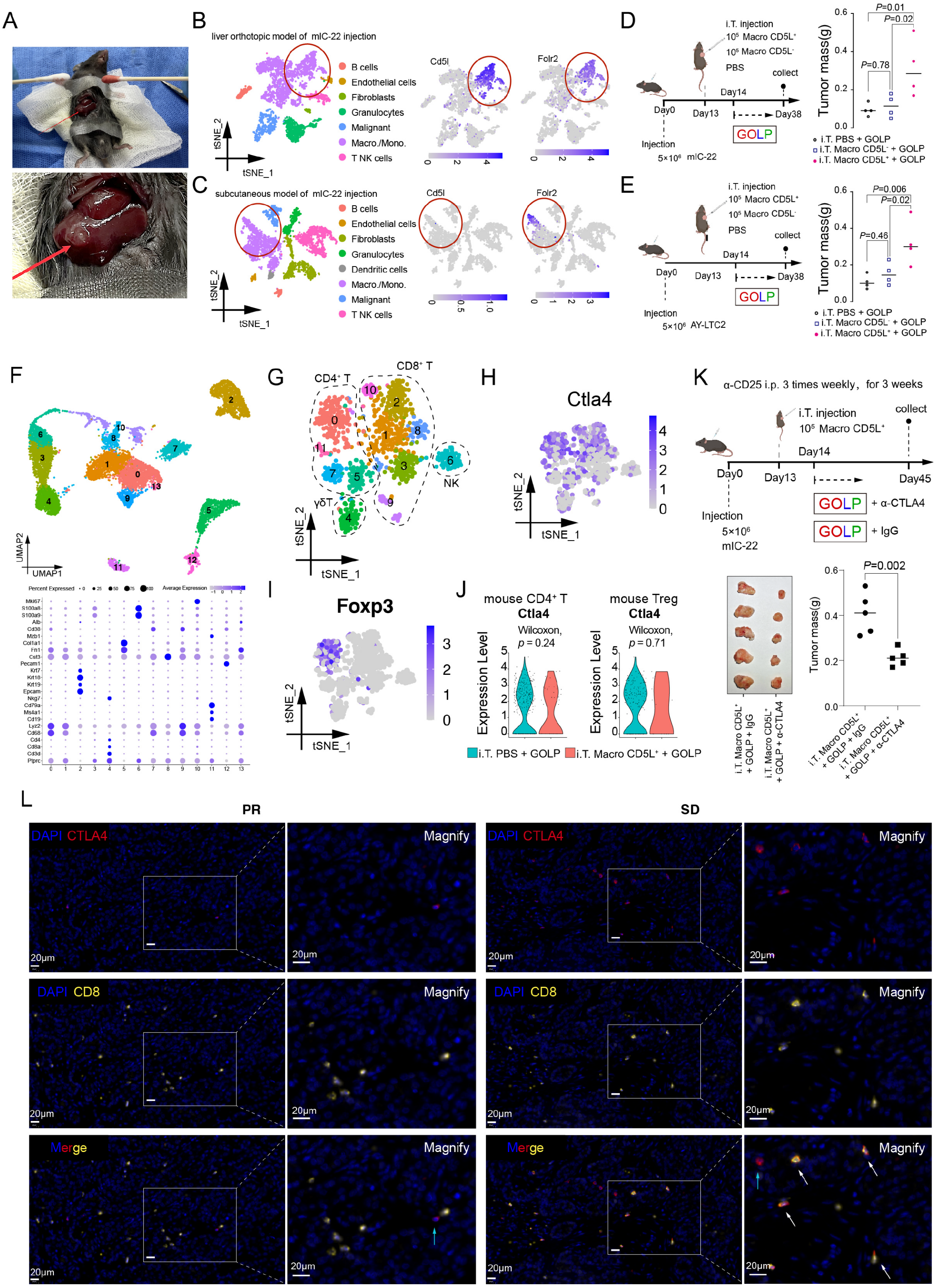

Supplementary Figure 8. Mouse iCCA models and CTLA4 expression in CD8$^+$ T of iCCA patients after GOLP treatment

(A) Construction of liver orthotopic injection model of mIC-22. t-test (two-sided), n=5 biologically independent animals for each group.

(B) scRNA analysis showing the major cell clusters and gene expression of Cd5l and Folr2 in the tumor

environment of liver orthotopic injection model (N =4,652 cells).

(C) scRNA analysis showing the major cell clusters and gene expression of Cd5l and Folr2 in the tumor environment of subcutaneous injection model (N =4,542 cells).

(D and E) Intra-tumoral injection of Macro CD5L^{-} or PBS showed no significant difference in affecting tumor response to GOLP therapy in mIC-22 or AY-LTC2 model. Macro CD5L^{+} injection resulted in significantly larger tumors mass compared to Macro CD5L^{-} or PBS control in mIC-22 or AY-LTC2 model (t test, two-sided, n=5 biologically independent animals for each group). Created with BioRender.com.

(F) Uniform manifold approximation and projection (UMAP) plot depicting 13 cell lineages in the subcutaneous injection model after GOLP treatment. Dot plot showing the average expression levels of key genes across the 13 cell types (N=32,285 cells).

(G) UMAP of lymphoid cells in mouse iCCA tumor (mIC-22) after GOLP therapy (N=1,129 cells).

(H and I) Feature plot showing the Ctla4 and Foxp3 expression in mouse lymphoid cells (N=1,129 cells).

(J) The expression level of Ctla4 in the mouse CD4^{+} T cells (N=374 cells) or Tregs (N=183 cells). Wilcoxon test, two-sided.

(K) Difference of tumor mass showed anti-CTLA4 treatment could still reverse Macro CD5L^{+}-induced GOLP resistance after depleting Tregs by anti-CD25 antibody. (t test, two-sided, N=5 biologically independent animals for each group). Created with BioRender.com.

(L) Original photos from Figure 8G showing representative samples (N=2 samples) from patients with PR or SD after GOLP stained by mIHC with anti-CD8 (yellow), anti-CTLA4 (red) antibodies. Scale bar, 20μm. Source data are provided as a Source Data file.

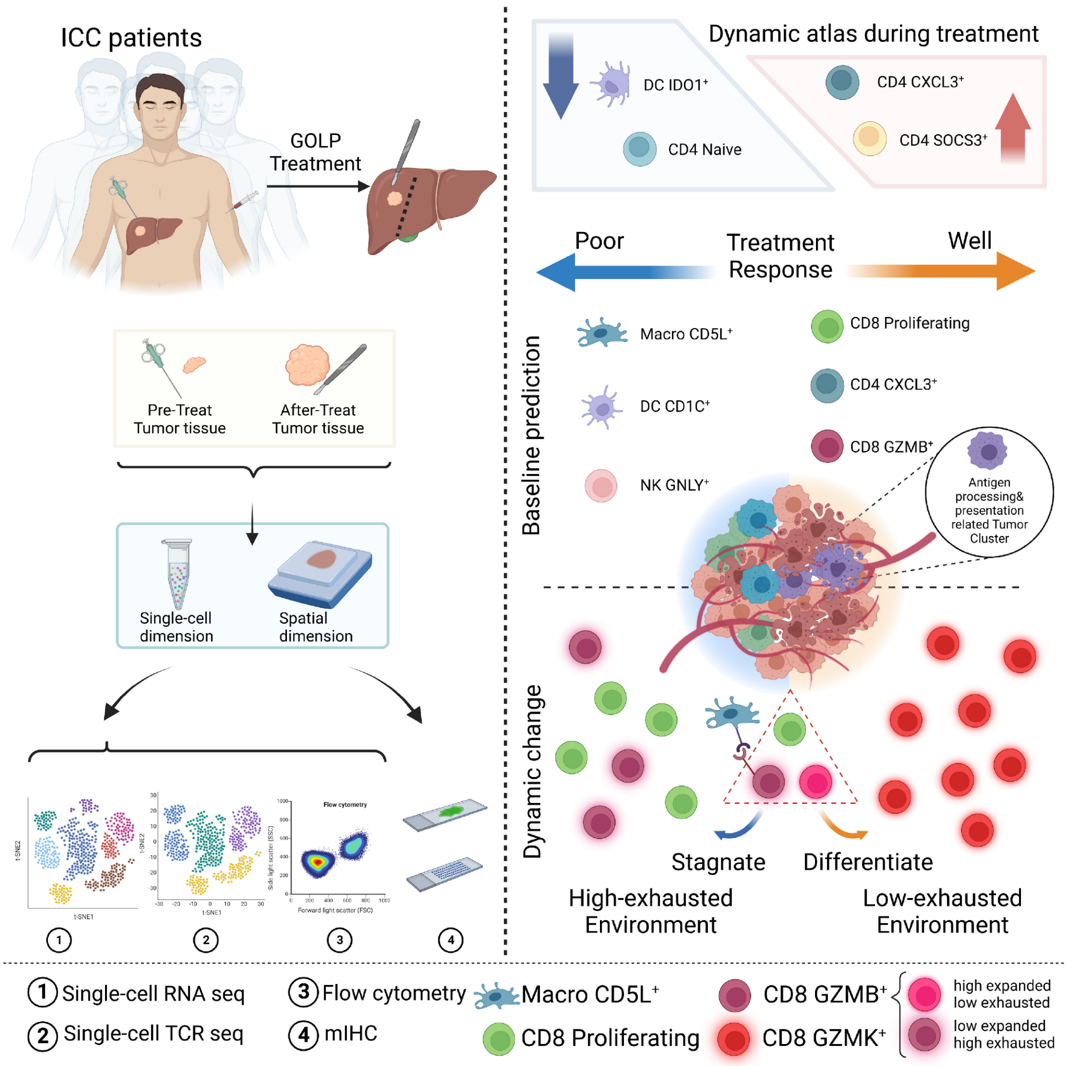

Supplementary Figure 9. Graphical abstract

This study characterizes the cellular and molecular dynamic atlas of the TME in patients with iCCA treated with the GOLP regimen. We identify the cell types responsible for sensitivity to GOLP treatment and the key cellular subtypes predicting therapeutic response in iCCA. Created with BioRender.com.
