# Supplementary material for: Macro CD5L+ deteriorates CD8+T cells exhaustion and impairs combination of Gemcitabine-Oxaliplatin-Lenvatinib-anti-PD1 therapy in intrahepatic cholangiocarcinoma
Source: Nat Commun. 2024 Jan 20;15:621. doi: 10.1038/s41467-024-44795-1 (PMC10799889; doi:10.1038/s41467-024-44795-1)
Supplement: Supplementary file 3 — Description of Additional Supplementary Files [file 41467_2024_44795_MOESM3_ESM.pdf]

**Description of Additional Supplementary Files**

Supplementary Data1. Cohort information.

Supplementary Data2. Analysis of main clusters.

Supplementary Data3. Analysis of tumor.

Supplementary Data4. Analysis of myeloid cells.

Supplementary Data5. Analysis of lymphoid cells.

Supplementary Data6. TCR and pseudotime.

Supplementary Data7. Celltalk analysis.

Supplementary Data8. Enriched TCR clone in pre- or post- GOLP treatment.

Supplementary Data9. Key resources table.
