# Supplementary material for: Macro CD5L+ deteriorates CD8+T cells exhaustion and impairs combination of Gemcitabine-Oxaliplatin-Lenvatinib-anti-PD1 therapy in intrahepatic cholangiocarcinoma
Source: Nat Commun. 2024 Jan 20;15:621. doi: 10.1038/s41467-024-44795-1 (PMC10799889; doi:10.1038/s41467-024-44795-1)
Supplement: Supplementary file 5 — Reporting Summary [file 41467_2024_44795_MOESM5_ESM.pdf]

nature portfolio

Corresponding author(s): Guo-Ming Shi, Jian Zhou, Yi-Di Sun, Jia Fan

Last updated by author(s): Dec 7, 2023

Reporting Summary

Nature Portfolio wishes to improve the reproducibility of the work that we publish. This form provides structure for consistency and transparency in reporting. For further information on Nature Portfolio policies, see our Editorial Policies and the Editorial Policy Checklist.

Statistics

For all statistical analyses, confirm that the following items are present in the figure legend, table legend, main text, or Methods section.

n/a	Confirmed	
☐	☒	The exact sample size (*n*) for each experimental group/condition, given as a discrete number and unit of measurement
☐	☒	A statement on whether measurements were taken from distinct samples or whether the same sample was measured repeatedly
☐	☒	The statistical test(s) used AND whether they are one- or two-sided<br>*Only common tests should be described solely by name; describe more complex techniques in the Methods section.*
☒	☐	A description of all covariates tested
☒	☐	A description of any assumptions or corrections, such as tests of normality and adjustment for multiple comparisons
☐	☒	A full description of the statistical parameters including central tendency (e.g. means) or other basic estimates (e.g. regression coefficient) AND variation (e.g. standard deviation) or associated estimates of uncertainty (e.g. confidence intervals)
☐	☒	For null hypothesis testing, the test statistic (e.g. *F*, *t*, *r*) with confidence intervals, effect sizes, degrees of freedom and *P* value noted<br>*Give P values as exact values whenever suitable.*
☒	☐	For Bayesian analysis, information on the choice of priors and Markov chain Monte Carlo settings
☒	☐	For hierarchical and complex designs, identification of the appropriate level for tests and full reporting of outcomes
☐	☒	Estimates of effect sizes (e.g. Cohen's *d*, Pearson's *r*), indicating how they were calculated

Our web collection on statistics for biologists contains articles on many of the points above.

Software and code

Policy information about availability of computer code

Data collection	Single-cell RNA and TCR V(D)J libraries were sequenced by an Illumina Novaseq6000 with 150 bp paired-end reads. All software and algorithms used in this study are listed in the section of Method.
Data analysis	The custom codes in this paper can be found on https://github.com/zhaodalv/GOLP_code.<br>The softwares used in this paper are listed as follow: Cellranger (v6.0.2); Scanpy (v1.8.2); Anndata(v0.7.8); Scrublet; Scirpy (v1.7.1); Monocle2 (v2.10.1); InferCNV (v1.6.0); NATMI (v2.0); CellChat (v1.1.3); pySCENIC (v0.11.2); SCENIC (v1.1.2); kneed (v0.7.0); SCSA; velocyto (v0.17.17); scVelo(0.2.4); PAGA; Metascape (v3.5); cNMF (v1.3); scikit-learn (v1.0); inferCNVpy (v2); survminer (v0.4.9); GSVA (v1.42.0); Seurat(v4.0); ggplot2 (v3.3.5). All softwares used in this manuscripts were described in the section of Method.

For manuscripts utilizing custom algorithms or software that are central to the research but not yet described in published literature, software must be made available to editors and reviewers. We strongly encourage code deposition in a community repository (e.g. GitHub). See the Nature Portfolio guidelines for submitting code & software for further information.

Data

Policy information about availability of data

All manuscripts must include a data availability statement. This statement should provide the following information, where applicable:

- Accession codes, unique identifiers, or web links for publicly available datasets
- A description of any restrictions on data availability
- For clinical datasets or third party data, please ensure that the statement adheres to our policy

Raw sequencing data of scRNA and TCR generated in this study have been deposited in the National Genomics Data Center with the accession code (PRJCA021882) [https://ngdc.cncb.ac.cn/bioproject/browse/PRJCA021882], with a copy deposition in biosino NODE database (OEP003206) [https://www.biosino.org/node/project/detail/OEP003206]. Data are available upon request through the repository portal. The raw sequencing data is only available for non-commercial and academic purposes under controlled access due to ethical and legal restrictions. The corresponding authors will respond to requests for the data in two weeks. The data will be available for three months once access has been granted. The publicly available data of bulkRNA and clinicopathologic information used in this study are available in the Supplemental information (Table S1) of the paper [https://www.cell.com/cancer-cell/fulltext/S1535-6108(21)00659-0#supplementaryMaterial]69. The cisTarget Human motif database used by SCENIC method88 in this study are available in https://resources.aertslab.org/cistarget/motif2tf/motifs-v9-nr.flybase-m0.001-o0.0.tbl. The remaining data are available within the Article, Supplementary Information or Source Data file. Source data are provided with this paper.

Research involving human participants, their data, or biological material

Policy information about studies with human participants or human data. See also policy information about sex, gender (identity/presentation), and sexual orientation and race, ethnicity and racism.

Reporting on sex and gender	We did not perform analysis based on sex and gender in our study. Both females and males were enrolled in our study without any prejudice.
Reporting on race, ethnicity, or other socially relevant groupings	No particular attention given to race, ethnicity, or other socially pertinent factors.
Population characteristics	The characteristics of the enrolled patients were listed in Supplementary Data1.
Recruitment	A total of 55 patients with GOLP treatment in the two cohorts were enrolled in this study (27 patients in NCT03951597, 28 patients in FDU-ZS-iCCA-T). Another 591 patients in the two GOLP treatment-naive cohorts,including 262 patients in FU-iCCA (Cancer Cell. 2022;40:70-87.e15) and 329 patients in FDU-ZS-iCCA were also enrolled into this study. All the patients were diagnosed with iCCA pathologically in Zhongshan Hospital, Fudan University. Detailed clinical information of the patients was showed in Supplementary Data1.
Ethics oversight	Written informed consent for clinical information collection and tissue collection was obtained from all the patients, and the collection of human samples and clinical information was approved by the Zhongshan Hospital Research Ethics Committee

Note that full information on the approval of the study protocol must also be provided in the manuscript.

Field-specific reporting

Please select the one below that is the best fit for your research. If you are not sure, read the appropriate sections before making your selection.

☒ Life sciences ☐ Behavioural & social sciences ☐ Ecological, evolutionary & environmental sciences

For a reference copy of the document with all sections, see nature.com/documents/nr-reporting-summary-flat.pdf

Life sciences study design

All studies must disclose on these points even when the disclosure is negative.

Sample size	The sample size is showed in the Figure Legends or in the Figures. The sample sizes employed in this study are consistent with previously published papers in Nature communications (Nat Commun 14, 8175 (2023). Nat Commun 14, 7863 (2023))
Data exclusions	No data was excluded.
Replication	All samples represent a minimum of three replicates and the attempts at replication were successful.
Randomization	Animals in this experiment were randomly assigned to different groups before treatment.
Blinding	Blinding was not performed during animal treatment in this study since lenvatinib showed obviously different color to other regimens. The investigators were blinded to group allocation during data collection and analysis.

Reporting for specific materials, systems and methods

We require information from authors about some types of materials, experimental systems and methods used in many studies. Here, indicate whether each material, system or method listed is relevant to your study. If you are not sure if a list item applies to your research, read the appropriate section before selecting a response.

Materials & experimental systems

n/a	Involved in the study
	☒ Antibodies
	☒ Eukaryotic cell lines
☒	☐ Palaeontology and archaeology
	☒ Animals and other organisms
	☒ Clinical data
☒	☐ Dual use research of concern
☒	☐ Plants

Methods

n/a	Involved in the study
☒	☐ ChIP-seq
	☒ Flow cytometry
☒	☐ MRI-based neuroimaging

Antibodies

Antibodies used

FITC anti-human Granzyme K (Biolegend # 370508, dilution 1:100)
APC/PercP-Cy5.5 anti-human CD8 (Biolegend #344721/344709, dilution 1:200)
BV421 anti-human CD233(LAG-3) (Biolegend # 369313, dilution 1:100)
BV421 anti-human CD161 (Biolegend #339913, dilution 1:100)
BV421 anti-human CD152(CTLA4) (Biolegend #369605, dilution 1:100)
PE anti-human CD279 (PD-1) (Biolegend # 135205, dilution 1:100)
Brilliant Violet 510™ anti-human Ki-67 (Biolegend #350518, dilution 1:100)
Alexa Fluor® 700/FITC anti-human CD45 (Biolegend #368514/103107, dilution 1:200)
APC/PE anti-human FOLR2 (Biolegend #391705/391703, dilution 1:100)
APC/PE anti-mouse Folate Receptor β (FR-β) (Biolegend #153305/153303, dilution 1:100)
PerCP anti-mouse F4/80 (Biolegend #123125, dilution 1:200)
FITC anti-mouse CD45 (Biolegend #157607, dilution 1:200)
PE anti-human/mouse Granzyme B (Biolegend # 372207, dilution 1:100)
Anti-CTLA4 antibody (Abcam #ab237712, dilution 1:500)
Anti-CD5L/CT-2 antibody (Abcam #ab45408, dilution 2μg/mL)
Anti-Granzyme K antibody (Abcam #ab282703, dilution 1:250)
Anti-Granzyme B antibody (Abcam #ab4059, dilution 1:500)
Anti-CD8 alpha antibody (Abcam #ab217344, dilution 1:300)
Anti-CK19 antibody (Abcam #ab133496, dilution 1:500)
Anti-CD68 antibody (Gene Tech (Shanghai) Company Limited # GM087607, dilution 1:400)

Validation

All antibodies were all commercial and have been confirmed by manufacturers.
FITC anti-human Granzyme K (Biolegend #370508) was confirmed by manufacturer (https://www.biolegend.com/en-us/products/fitc-anti-human-granzyme-k-antibody-13860)
APC/PercP-Cy5.5 anti-human CD8 (Biolegend #344721/344709) was confirmed by manufacturer (https://www.biolegend.com/en-us/products/apc-anti-human-cd8-antibody-6531;https://www.biolegend.com/en-us/products/percp-cyanine5-5-anti-human-cd8-antibody-6389)
BV421 anti-human CD233(LAG-3) (Biolegend #369313) was confirmed by manufacturer (https://www.biolegend.com/en-us/products/brilliant-violet-421-anti-human-cd223-lag-3-antibody-13668)
BV421 anti-human CD161 (Biolegend #339913) was confirmed by manufacturer (https://www.biolegend.com/en-us/products/brilliant-violet-421-anti-human-cd161-antibody-7546)
BV421 anti-human CD152(CTLA4) (Biolegend #369605) was confirmed by manufacturer (https://www.biolegend.com/en-us/products/brilliant-violet-421-anti-human-cd152-ctla-4-antibody-13159)
PE anti-human CD279 (PD-1) (Biolegend #135205) was confirmed by manufacturers (https://www.biolegend.com/en-us/products/pe-anti-mouse-cd279-pd-1-antibody-6170)
Brilliant Violet 510™ anti-human Ki-67 (Biolegend #350518) was confirmed by manufacturer (https://www.biolegend.com/en-us/products/brilliant-violet-510-anti-human-ki-67-antibody-8571)
Alexa Fluor® 700/FITC anti-human CD45 (Biolegend #368514/103107) was confirmed by manufacturers (https://www.biolegend.com/en-us/products/alexa-fluor-700-anti-human-cd45-antibody-12399; https://www.biolegend.com/en-us/products/fitc-anti-mouse-cd45-antibody-99)
APC/PE anti-human FOLR2 (Biolegend #391705/391703) was confirmed by manufacturers (https://www.biolegend.com/en-us/products/apc-anti-human-folate-receptor-beta-fr-beta-antibody-15117; https://www.biolegend.com/en-us/products/pe-anti-human-folate-receptor-beta-fr-beta-antibody-15115)
APC/PE anti-mouse Folate Receptor β (FR-β) (Biolegend #153305/153303) was confirmed by manufacturer (https://www.biolegend.com/en-us/products/apc-anti-mouse-folate-receptor-beta-fr-beta-antibody-15144; https://www.biolegend.com/en-us/products/pe-anti-mouse-folate-receptor-beta-fr-beta-antibody-15147)
PerCP anti-mouse F4/80 (Biolegend #123125) was confirmed by manufacturer (https://www.biolegend.com/en-us/products/percp-anti-mouse-f4-80-antibody-4302)
FITC anti-mouse CD45 (Biolegend #157607) was confirmed by manufacturer (https://www.biolegend.com/en-us/products/fitc-anti-mouse-cd45-recombinant-antibody-18940)
PE anti-human/mouse Granzyme B (Biolegend #372207) was confirmed by manufacturer (https://www.biolegend.com/en-us/

products/pe-anti-human-mouse-granzyme-b-recombinant-antibody-14431)
Anti-CTLA4 antibody (Abcam #ab237712) was confirmed by manufacturer (https://www.abcam.cn/products/primary-antibodies/ctla4-antibody-cal49-ab237712.html)
Anti-CD5L/CT-2 antibody (Abcam #ab45408) was confirmed by manufacturer (https://www.abcam.cn/products/primary-antibodies/cd5lct-2-antibody-ab45408.html)
Anti-Granzyme K antibody (Abcam #ab282703) was confirmed by manufacturer (https://www.abcam.cn/products/primary-antibodies/granzyme-k-antibody-epr24601-164-ab282703.html)
Anti-Granzyme B antibody (Abcam #ab4059) was confirmed by manufacturer (https://www.abcam.cn/products/primary-antibodies/granzyme-b-antibody-ab4059.html)
Anti-CD8 alpha antibody (Abcam #ab217344) was confirmed by manufacturer (https://www.abcam.cn/products/primary-antibodies/cd8-alpha-antibody-epr21769-ab217344.html)
Anti-CK19 antibody (Abcam #ab133496) was confirmed by manufacturer (https://www.abcam.cn/products/primary-antibodies/cytokeratin-19-antibody-epncir127b-ab133496.html)
Anti-CD68 antibody (Gene Tech (Shanghai) Company Limited #GM087607) was confirmed by manufacturer (https://www.genetech.com.cn/goods/goods_detail/269.html)

Eukaryotic cell lines

Policy information about cell lines and Sex and Gender in Research

Cell line source(s)	In this study, we used mouse intrahepatic cholangiocarcinoma cell line mIC-22, which was developed from a male C57/BL 6J mice in our laboratory. Another mouse cell line AY-LTC2 is a gift from Liu's Lab.
Authentication	Tumorigenicity and IHC identification of the cell lines mIC-22 and AY-LTC2 can be found in FigureS1 F and H.
Mycoplasma contamination	The cells in the experiment were free of mycoplasma contamination.
Commonly misidentified lines (See ICLAC register)	None of the cell lines used was listed in the database of ICLAC.

Animals and other research organisms

Policy information about studies involving animals; ARRIVE guidelines recommended for reporting animal research, and Sex and Gender in Research

Laboratory animals	C57BL/6J mice (male, 5-6 week-old) were used in our study. Detailed information for animal study can be found in the section of Method. Temperatures of 18-25°C with 50-60% humidity and 14-hour light/10-hour dark cycle are used.
Wild animals	No wild animals were used.
Reporting on sex	Male mice were used in our study to confirm the findings from human samples, since the mIC-22 cell line was developed by a male C57BL/6J. The findings from human samples were not limited to one sex.
Field-collected samples	No field-collected samples were used.
Ethics oversight	All animal procedures were reviewed and approved by the Zhongshan Hospital Research Ethics Committee.

Note that full information on the approval of the study protocol must also be provided in the manuscript.

Clinical data

Policy information about clinical studies
All manuscripts should comply with the ICMJEguidelines for publication of clinical research and a completedCONSORT checklist must be included with all submissions.

Clinical trial registration	This study is not a clinical trial.
Study protocol	This study is not a clinical trial.
Data collection	This study is not a clinical trial.
Outcomes	This study is not a clinical trial and do not set outcomes.

Plants

Seed stocks	This study does not involve plants.
Novel plant genotypes	This study does not involve plants.
Authentication	This study does not involve plants.

Flow Cytometry

Plots

Confirm that:

- [x] The axis labels state the marker and fluorochrome used (e.g. CD4-FITC).
- [x] The axis scales are clearly visible. Include numbers along axes only for bottom left plot of group (a 'group' is an analysis of identical markers).
- [x] All plots are contour plots with outliers or pseudocolor plots.
- [x] A numerical value for number of cells or percentage (with statistics) is provided.

Methodology

Sample preparation	Isolated single-cell suspensions from the tissue were centrifuged (350Xg, 6min, 4°C) and resuspended in 100 µl Cell Staining Buffer (Biolegend, Cat.420201). Human TruStain FcX™ (Fc Receptor Blocking Solution, BioLegend Cat. No. 422301) was used to incubate with the single-cell suspensions for 15 min at room temperature before cell-surface staining.
Instrument	Beckman MoFlo XDP; BD LSRFortessa™ X-20 Flow Cytometer
Software	FlowJo 10.6.2
Cell population abundance	30000 cells were sorted per sample. Positive and negative boundaries were determined by the unstain control.
Gating strategy	Positive and negative boundaries were determined by the unstain control (see Figure S5F)

- [x] Tick this box to confirm that a figure exemplifying the gating strategy is provided in the Supplementary Information.
